# BMI Fails to Reflect the Developmental Changes in Body Fatness between Boys and Girls during Adolescence

**DOI:** 10.3390/ijerph18157833

**Published:** 2021-07-23

**Authors:** Alan M. Nevill, Cézane Priscila Reuter, Caroline Brand, Anelise Reis Gaya, Jorge Mota, Jane Dagmar Pollo Renner, Michael J. Duncan

**Affiliations:** 1 Faculty of Education, Health and Wellbeing, University of Wolverhampton, Walsall WS1 3BD, UK; a.m.nevill@wlv.ac.uk; 2 Graduate Program in Health Promotion, University of Santa Cruz do Sul, Santa Cruz do Sul 96816-501, Brazil; cezanereuter@unisc.br (C.P.R.); carolbrand@hotmail.com.br (C.B.); janerenner@unisc.br (J.D.P.R.); 3 School of Physical Education, Physiotherapy and Dance, Federal University of Rio Grande do Sul, Porto Alegre 90040-060, Brazil; anegaya@gmail.com; 4 Faculty of Sport, University of Porto, 4099-002 Porto, Portugal; jmota@fade.up.pt; 5 Sport, Exercise and Life Sciences Research Centre, Coventry University, Coventry CV1 5FB, UK

**Keywords:** obesity, overweight, paediatrics, growth, anthropometry

## Abstract

Body mass index (BMI) is thought to reflect excess adiposity in both youth and adults alike. However, the association between BMI and fatness varies, especially as children grow into adults. Thus, the present study sought to address this issue by characterizing how BMI reflects age and sex differences in body fatness in 7–16-year-old children. Methods: This cross-sectional study was conducted with 2150 children and adolescents, aged 7 to 16 years from the city of Santa Cruz do Sul, Brazil. BMI (kg/m^2^), and percentage body fat, using tricipital and subscapular folds, were assessed. For statistical analysis, ANOVA and ANCOVA were used. Results: When considered in isolation, there was no significant interaction in the age-by-sex differences in BMI (*p* = 0.69). However, when we controlled for percent body fatness, the analysis revealed considerable age-by-sex differences in BMI (*p* < 0.001). Conclusion: For the same body fat (%), there are no differences in BMI in children <10 years.

## 1. Introduction

Overweight and obesity during childhood remain a worldwide health concern with over 340 million children and adolescents being classified as overweight or obese worldwide [1]. The negative health consequences of overweight and obesity are well established, and as a consequence, monitoring of children’s weight status alongside preventive approaches to reduce overweight and obesity are prevalent across the globe [2]. A key tenet of such monitoring approach and preventive approaches has been the use of body mass index (BMI) as the primary means by which overweight and obesity is classified. BMI remains the most frequently used proxy of obesity in epidemiological studies in both healthy and diseased populations [3]. It is also the recommended metric for assessing overweight and obesity advocated by the World Health Organisation [1]. Despite the proliferation and the convenient nature of assessing BMI, there are considerable known limitations of the use of BMI to assess overweight and obesity (see [4,5] for in-depth overview of this area).

Our principal concern is that BMI does not actually reflect body composition, rather it assesses weight for height, and the assertion that BMI reflects excess adiposity in childhood and adolescence is erroneous. For example, independent of body fatness, children and adolescents with shorter legs for their height will have higher BMI values compared with children with longer leg lengths relative to their height [6]. Data also suggest that for children with overweight and obesity, BMI is a poor predictor of percent body fatness [7]. This is not a new issue, and the shortcomings of BMI and BMI-related cut points for adults have been well demonstrated by Nevill and Metsios [3] who demonstrated that BMI did not reflect the same levels of adiposity in adults and that younger adults had greater BMI than older people for the same levels of adiposity, with differences ranging from 4 and 3 BMI units for males and females, respectively. Nevill and Metsios [3] concluded that utilisation of BMI in evidence-based approaches related to dietary or exercise intervention and clinical decision making for adult health needs to be reconsidered and, adjusted where appropriate to properly consider body fatness and to ensure BMI better reflects what it is purported to assess. This issue is particularly important when considering children and adolescents, as there are a number of physiological changes that occur during childhood and through adolescence, including rapid increases in physical size, hormonal fluctuations, changes in body composition and the timing of various maturational landmarks, which all influence adiposity (see [6] for a review). BMI is not independent of stature in children and is sensitive to body build with the magnitude of association between BMI and adiposity differing depending on age, sex and stage of development. Prior research has reported strong relationships between BMI and percent body fatness (all *r* > 0.8) in children aged 9–11 years of age from multiple countries across the world [8]. Despite this, Widhalm et al. [9] have observed that in children aged 6–17 years, the strongest relationship between BMI and body fatness was strongest (*r* = 0.79) in children aged 6–9 years, and that after this point, the strength of association declines, and importantly, significantly poorer relationships are seen in children and adolescents at the lower and upper ends of BMI after this age. This results in considerable question marks over the use of BMI, including the use of BMI for age cut points, to assess overweight and obesity in children without reconsidering or adjusting for body fatness. While Nevill and Metsios [3] have addressed this issue in adults, to date, this does not appear to have been considered in children. Any difference in BMI, after adjusting for body fatness, during childhood and adolescence has not yet been established. The present study sought to address this issue by characterising how BMI reflects age and sex differences in body fatness in 7–16 year old children.

## 2. Materials and Methods

This cross-sectional study was conducted with 2150 children and adolescents, aged between 7 to 16 years from the city of Santa Cruz do Sul, Brazil using data from a school based regional study, which has been running since 2004. Students of all regions of the city were considered to calculate the population density of students to be included in the research. Twenty-five schools were randomly selected from 50 schools with 20,380 schoolchildren. In 2004, all students from the 25 schools were invited to participate to form a cohort, and the evaluations were carried out in the following phases and years: Phase I (2004–2005), Phase II (2007–2009), Phase III (2011–2012), Phase IV (2014–2015) and Phase V (2016–2017). Data from Phase V were used for the present study as the most recent dataset available. The present study was approved by the research ethics committee at the University of Santa Cruz do Sul (no. 1.498.305), and it was conducted in accordance with Resolution 466/2012 of the National Council of Health in Brazil. The schoolchildren’s parents or legal guardians signed free and informed consent forms.

To be included in the sample of the present study, participants’ parents or guardians should have provided informed consent, and individuals aged ≥12 years should have signed the consent form. Adolescents who had not completed data on anthropometric evaluations were excluded from the study. Participants were eligible to participate if they were from one of the selected school, were aged between 7–16 years old and had no special educational need, musculoskeletal or cognitive impairment that prevented participation.

All variables analysed in the present study were measured at University of Santa Cruz do Sul by trained researchers. Height and weight were evaluated on the anthropometric scale with a coupled stadiometer (Filizola^®^, São Paulo, Brazil). Body mass index (BMI) was calculated by dividing body mass (in kilograms) by height (in square meters). To determine body fat percentage (%BF), tricipital and subscapular folds were measured using a Lange^®^ caliper (Beta Technology, Santa Cruz, CA, USA), and the equation of Slaughter et al. was applied [10]. This prediction equation has been shown as reliable and valid for the prediction of BF% in children [11,12] including Brazilian children and adolescents aged from 5–17 years [13,14]. The Slaughter et al. [10] equation is one of the most widely used prediction equations for the determination of BF% in children [13]. Descriptive data by sex and age are shown in Table 1.

### Statistical Methods

Differences in BMI and %BF were analysed using 2-way (sex-by-age) ANOVAs. Differences in BMI by age and sex (as fixed factors) controlling for difference in BF% were analysed using ANCOVA (taking BF% as the covariate). Recognising that BMI is positively skewed and unlikely to be normally distributed, we adopted a simple inverse transformation, known to overcome this problem [5,15]. A re-analysis of iBMI was conducted to confirm the divergent nature of the sex-by-age interaction (see Results).

## 3. Results

**Figure 1:**
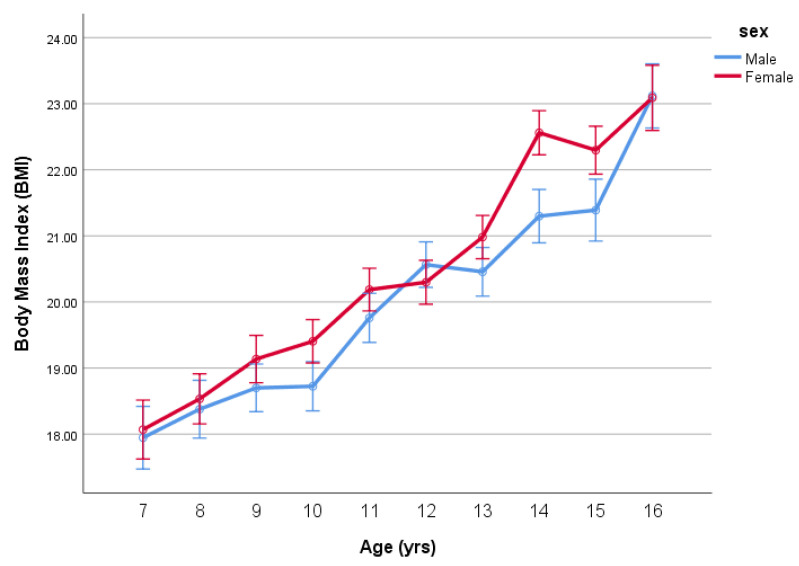
Mean BMI (mean ± SE) by age and sex.

ANOVA revealed differences in BMI due to age (*p* < 0.001), sex (*p* = 0.016) but no age-by-sex interaction (*p* = 0.69). The means for BMI (mean ± SE) by age and sex are given in Table 1 and Figure 1.

**Figure 2:**
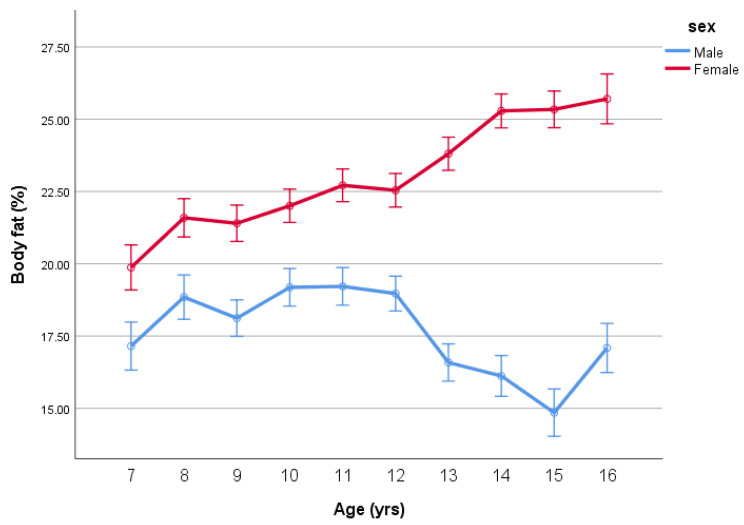
Body fat percentage (BF%) (mean ± SE) by age and sex.

However, these BMI means do not accurately reflect the age-by-sex differences in BF%. ANOVA revealed differences in BF% due to age (*p* = 0.022), sex (*p* < 0.001) and an age-by-sex interaction (*p* < 0.001), as given by Table 1 and Figure 2.

**Figure 3:**
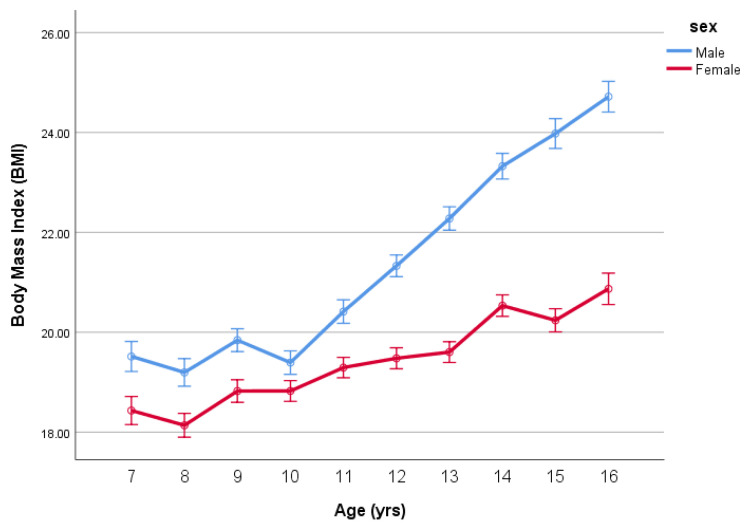
BMI (mean ± SE) by age and sex adjusted for BF%.

Indeed, when we analysed BMI by age and sex (as fixed factors) controlling for BF% using ANCOVA, the analysis revealed differences in both main effects age (*p* < 0.001), sex (*p* < 0.001) and the age-by-sex interaction (*p* < 0.001) (see Figure 3).

Clearly, for the same BF%, the boys mean BMIs increase steadily with age reflecting less BF% (more muscle mass) than girls as they go through puberty as seen in Figure 3. The mean differences in BMI (adjusted for BF%) by age between the boys and girls are given in Table 2. As children grow through puberty, the gap in BMI grows to over 4 BMI units by 16 years. These developmental changes in BMI emphasize a need to adjust BMI for body fatness to appropriately reflect overweight and obesity in children and adolescents.

BMI, similar to body mass, is known to be positively skewed and not normally distributed. This was confirmed when the residuals from the ANCOVA of BMI were saved and found to violate the normality assumption (see Table 3). A simple transformation (inverse BMI) is known to rectify this problem [5].

**Figure 4:**
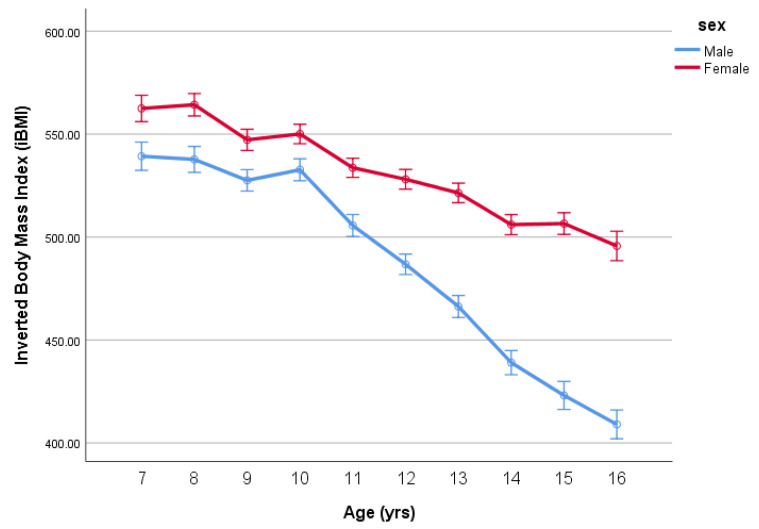
Inverted BMI (iBMI) (mean ± SE) by age and sex, adjusted for BF%.

When the ANCOVA was repeated using iBMI as the response variable, for the same BF%, the boys’ mean iBMIs declines steadily with age reflecting less BF% (more muscle mass) than the girls’ as they go through puberty (see Figure 4). As can be seen in Table 3, the residuals are much more normally distributed. The mean differences in iBMI (adjusted for BF%) by age between the boys and girls are given in Table 4 (note that the equivalent BMIs and sex differences are also reported). 

## 4. Discussion

The current study highlights important developmental trajectories of BMI and body fat changes during childhood and adolescence. While the limitations of BMI as a measure of obesity are well known, the present study highlights in a clear way the issues faced by public health professionals in the understanding and use of BMI in such situations. Our results demonstrate that when considered in isolation, there was no significant interaction in the age-by-sex differences in BMI in children and adolescents aged 7–16 years (see Figure 1). However, taking BMI in isolation hides an important issue if BMI is to be considered robust as a measure of overweight and obesity. In our subsequent analysis, when we controlled for percent body fatness, the analysis reveals considerable age-by-sex differences in BMI. The results of the present study, therefore, highlight that if we do not adjust for body fatness, BMI appears similar between sex groups, but if we account for body fatness, there is a considerable difference in BMI as children develop into adolescents.

**Figure 5:**
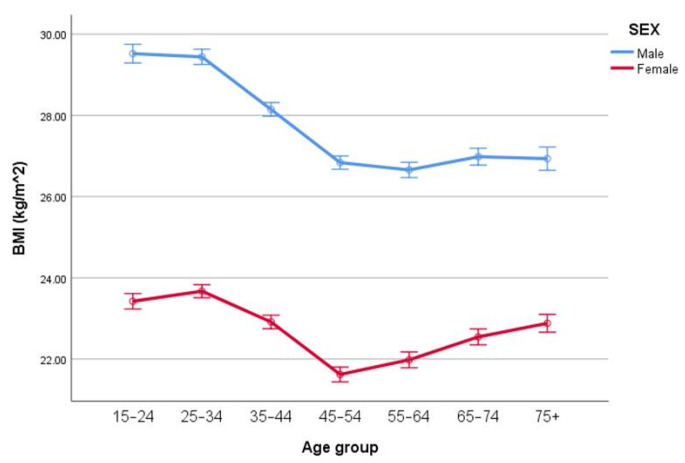
BMI (mean ± SE) by age and sex, adjusted for BF%, data provided by the authors from Nevill and Metsios [3].

This gap has been identified in an adult population [3], but the way in which any differences in BMI, after adjusting for body fatness, during childhood and adolescence has not. As a consequence, the present study extends our understanding in this area. The present study demonstrates that the divergence in BMI between boys and girls grows during adolescence as well as reinforcing the need to adjust for body fatness when using BMI in this population. The developmental trajectories we identify show a continuing divergence in BMI between boys and girls from the age of 11 to 16. Although maturation was not assessed in the current study, this divergence would broadly align with the onset of puberty in adolescents, with the physiological changes (e.g., increase in muscle mass in boys) associated with puberty explaining this divergence [16]. When the data in the current study are overlaid with adult data presented by Nevill and Metsios [3], the BMI gap identified at 16 years of age in the current study aligns directly with the BMI gap identified between males and females of the same age by Nevill and Metsios [3] (see Figure 5).

The argument is often made that the use of BMI persists because it is easier to assess, is time economical and not labour intensive, compared to the assessment of body fatness. While the data in the present study suggest it is important to adjust for body fatness when examining BMI in children and adolescents. In the present study, we also analysed the data using inverted BMI, which addresses many of the limitations of BMI yet is as easy to assess. Notably, the iBMI data demonstrate a similar pattern to that of the age-by-sex interaction for body fat adjusted BMI. iBMI is considered more a measure of lean mass and has a robust biological basis, unlike BMI (see [17] for a review). Importantly, iBMI is no more onerous or time consuming to assess than BMI and, thus, provides a practical and efficient way to assess weight status in children and adolescents, which is superior to BMI.

We are conscious not to overstate the findings of the current study, but identifying developmental trajectory of BMI through childhood and adolescence and the extent to which it reflects body fatness is an important consideration for public health, where the use of BMI is particularly commonplace. There are limitations of the current study including the cross sectional design and that the findings are only reflective of Brazilian children. We are conscious that the use of skinfolds to assess body fatness in the present study, although a valid and reliable field measure, may not provide as accurate an assessment of body fatness as measures such as multi frequency bioelectrical impedance analysis or dual X-ray absorptiometry. Such modes of assessment were not feasible in the large number of participants and context of the current study. However, predictive equations based on skinfolds have been reported to be a valid method for estimating body composition in epidemiological studies developed with children and adolescents, including the Slaughter et al. [10] prediction equation used in the current study [11,12]. The Slaughter et al. [10] equation considers constants adjusted by a quadratic model, indicating the sum of triceps and subscapular skinfold thicknesses as a good predictor of fat percentage, which takes into account maturational stage, sex, age and race. Therefore, although we recognize that equations are not the gold standard method for determining fat percentage, considering the large sample size of the present study, it is an appropriate and effective method. The equation [10] has been used in large scale studies such as the Bogalusa Heart study to predict %BF with children aged 5–17 [13] as well as being used in samples of Brazilian children previously [14]. Indeed, this is an exploratory study that does not aim to disregard the curves of the World Health Organisation that are used worldwide as a means for evaluating the nutritional profile and the direct comparison between absolute values of BMI and %BF could be considered a limitation. This study aimed to expand the use of BMI as an indicator of body fat, and it should be considered a first step in considering the influence of the percentage of body fat, noting that BMI together with the percentage of fat can be an important indicator of adiposity. Pubertal development and biological age are also other key considerations when considering the results of the current study. Due to the sensitive nature of assessment, establishing the stage of pubertal development was not undertaken in the current study. Body fatness is purported to influence timing of puberty [18], and the process of puberty, including growth spurts, moving from childhood to adolescence, can impact the strength of relationship between BMI and body fatness [6]. Instead our analysis is based on chronological age, and the results of the present study should, therefore, be interpreted in light of this. In future work, where constraints allow for assessing maturation may be beneficial in extending the findings of the current study.

## 5. Conclusions

The present study demonstrates a need to adjust BMI for body fatness if it is to appropriately reflect overweight and obesity in children and adolescents. The use of iBMI offers an equally time and labour-efficient method as BMI but offers benefits in having a sound biological basis and accurately reflecting lean mass, which can accurately reflect the changes in body fatness across childhood and adolescence.

## Figures and Tables

**Table 1:** Anthropometric descriptive data for the sample.

Sex	Age	N	Stature	SD	Mass	SD	BMI	SD	%Fat	SD
Male	7	68	1.29	0.06	30.08	7.16	17.95	3.29	17.15	7.64
	8	80	1.33	0.07	32.72	7.29	18.38	2.86	18.85	6.73
	9	118	1.37	0.07	35.41	8.52	18.70	3.74	18.12	7.33
	10	111	1.42	0.07	38.03	8.82	18.72	3.10	19.19	8.01
	11	111	1.47	0.08	43.27	10.34	19.76	3.44	19.22	8.00
	12	129	1.54	0.09	49.31	12.12	20.57	4.09	18.97	7.95
	13	113	1.60	0.10	53.05	15.87	20.46	4.48	16.58	8.20
	14	94	1.67	0.10	59.93	14.46	21.30	4.10	16.12	7.05
	15	70	1.71	0.09	62.66	13.43	21.39	3.84	14.85	7.30
	16	65	1.73	0.08	69.13	14.83	23.12	4.08	17.09	7.01
	Total	959	1.51	0.16	46.65	16.50	19.95	3.99	17.79	7.70
Female	7	77	1.27	0.07	29.26	7.97	18.07	3.45	19.87	6.70
	8	107	1.32	0.08	32.61	8.57	18.53	3.39	21.59	6.50
	9	119	1.38	0.07	36.87	9.44	19.14	3.73	21.40	6.45
	10	142	1.44	0.07	40.29	10.40	19.40	4.25	22.00	6.49
	11	147	1.49	0.08	45.46	11.57	20.19	4.01	22.71	6.13
	12	139	1.53	0.08	48.06	11.43	20.30	3.98	22.54	6.11
	13	143	1.58	0.07	52.31	10.26	20.98	3.70	23.81	5.69
	14	137	1.61	0.07	58.12	13.44	22.56	5.12	25.29	6.07
	15	117	1.60	0.07	57.10	9.38	22.30	3.83	25.34	5.49
	16	63	1.62	0.07	60.32	12.05	23.09	4.34	25.70	6.47
	Total	1191	1.49	0.13	46.30	14.31	20.45	4.29	23.03	6.38

BMI, body mass index.

**Table 2:** The mean differences in BMI (adjusted for BF%) by age between the boys and girls.

Age	BMI Male	BMI Female	Difference between Male and Female
7	19.5	18.4	1.1
8	19.2	18.1	1.1
9	19.8	18.8	1.0
10	19.4	18.8	0.6
11	20.4	19.3	1.1
12	21.3	19.5	1.9
13	22.3	19.6	2.7
14	23.3	20.5	2.8
15	24.0	20.2	3.7
16	24.7	20.9	3.8

**Table 3:** Normality Data.

	Tests of Normality					
	Kolmogorov–Smirnov ^a^			Shapiro–Wilk		
	Statistic	df	Sig.	Statistic	df	Sig.
Residual for BMI	0.048	2149	0.000	0.959	2149	0.000
Residual for iBMI	0.018	2149	0.086	0.997	2149	0.001

^a^ Lilliefors significance correction; BMI, body mass index; iBMI, inverse body mass index, df, degrees of freedom; Sig., significance.

**Table 4:** The mean differences in iBMI (adjusted for BF%) by age between the boys and girls are given in Table 4 (note that the equivalent BMIs and sex differences are also reported).

Age	iBMI					BMI				
	Male	SEE	Female	SEE	Diff	Male	SEE	Female	SEE	Diff
7	539.2	6.9	562.5	6.4	23.2	18.5	0.30	17.8	0.28	0.8
8	537.7	6.3	564.3	5.4	26.6	18.6	0.28	17.7	0.24	0.9
9	527.5	5.2	547.2	5.2	19.7	19.0	0.23	18.3	0.23	0.7
10	532.7	5.3	550.1	4.7	17.4	18.8	0.23	18.2	0.21	0.6
11	505.6	5.3	533.6	4.7	28.0	19.8	0.23	18.7	0.20	1.0
12	486.8	5.0	528.1	4.8	41.3	20.5	0.22	18.9	0.21	1.6
13	466.3	5.3	521.4	4.7	55.2	21.4	0.23	19.2	0.21	2.3
14	439.0	5.9	506.0	4.9	67.0	22.8	0.26	19.8	0.21	3.0
15	423.1	6.8	506.6	5.3	83.5	23.6	0.30	19.7	0.23	3.9
16	409.0	7.0	495.7	7.1	86.7	24.4	0.31	20.2	0.31	4.3

SEE, Standard Error of Estimate.

## Data Availability

The data presented in this study are available on request from the corresponding author.
